# Commentary: The wars within – uncomfortable truths about the bullying of girls in conflict‐affected societies

**DOI:** 10.1111/camh.70006

**Published:** 2025-07-02

**Authors:** Mina Fazel

**Affiliations:** ^1^ Department of Psychiatry University of Oxford Oxford UK; ^2^ Psychological Medicine The Children's Hospital, Oxford University Hospitals NHS Foundation Trust Oxford UK

**Keywords:** Bullying victimization, adolescent development, armed conflict, post‐conflict settings, gender‐based aggression

## Abstract

This commentary reflects on a timely and methodologically significant study by Silwal et al., which investigates bullying victimization among adolescents in conflict‐affected Eastern Ukraine. Conducted in a context of fragility, social fragmentation, and resource scarcity, the study offers vital insights into how war and its aftermath shape adolescent experiences. It reveals higher rates of bullying in conflict‐affected regions, with girls disproportionately targeted—an uncomfortable finding that challenges conventional gender patterns in bullying. Drawing on emerging evidence, the commentary considers the role of desensitization, emotional regulation, and digital exposure in shaping youth aggression. It also highlights the need to address the structural stressors facing adolescents in both post‐conflict and post‐migration contexts, particularly within disrupted school systems. In response, the commentary calls for integrated and context‐responsive interventions that strengthen emotional and social competencies without reinforcing stigma. It further urges researchers and policymakers to acknowledge the politicized nature of post‐migration violence discourse and to maintain commitment to nuanced and context‐sensitive analysis. The findings underscore that bullying in such settings is a social indicator of wider systemic pressures—an expression of the hidden wars adolescents carry in their daily lives and into their schools.

Conducting empirical research in conflict‐affected settings is immensely challenging. Studies such as that by Silwal et al. conducted in 2016–2017, following the onset of conflict in Eastern Ukraine, are rare, but vital (Silwal et al., 2025). The fragility and volatility of the post‐conflict environment, combined with social fragmentation and resource scarcity, complicate data collected – particularly when humanitarian priorities are pressing. Therefore, this study investigating bullying victimization among Ukrainian adolescents in both a post‐conflict community in Eastern Ukraine and another relatively unaffected area, is especially commendable. It offers important insights into the intersectionality of adolescence, gender, and geopolitical instability and highlights the importance of remaining vigilant about the interpersonal consequences of war–even when ‘peace,’ however fragile, has been restored.

The researchers found that adolescents in the war‐affected region experienced higher rates of bullying, and the girls, in particular, were disproportionately targeted. This study brings to light uncomfortable truths about the enduring and multifaceted impacts of war. War does not merely destroy infrastructure and displace populations; its effects at the individual level extend beyond an increased risk of the most severe mental illnesses to also encompass disruptions in emotional regulation, interpersonal relationships, and the shaping of social values (Abdi et al., 2023). The study thus highlights three critical areas for academic inquiry: how to better understand post‐conflict contexts, given the methodological and logistical challenges of conducting research; the impact of increasing exposures to violence on adolescent development; and the disproportionate vulnerability of girls and women to interpersonal harm not only peri‐ but also post‐conflict. Understanding these factors is essential for identifying effective strategies and designing interventions that are appropriately tailored to the needs of post‐conflict populations–both in countries of origin and in post‐migration settings.

The findings underscore an important point: in conflict‐affected settings, adolescents are not only exposed to organized violence – whether directly, as witnesses or through indirect means – but also encounter it in homes, schools, and increasingly, in digital spaces. As such, there is a danger that, for some, violence becomes commonplace. This leads us to ask: does exposure to traumatic events across multiple domains lead to desensitization, whereby aggression becomes normalized and potentially perceived as an acceptable form of conflict resolution?

A growing body of literature links various forms of violence exposure to maladaptive aggressive behavior in youth. Walters (2021) emphasizes that bullying victimization can itself be a precursor to perpetration, highlighting the continuity from early victimization to later aggressive conduct. Other studies highlight the range of negative adolescent outcomes following violence exposure (Jagasia et al., 2024). More recently, some have argued that digital exposures – such as online aggression and violent immersive content – impact adolescents with Chen, Ho, and Lwin (2020) demonstrating that exposure to online aggression can predict both victimization and perpetration in cyberbullying.

In conflict‐affected settings, where social and institutional containment is weakened, exposures that might reinforce aggression may be particularly harmful (Schmitt et al., 2022). Until we better understand whether certain developmental periods confer greater sensitivity to aggressive experiences or observations, we should remain cautious. Adolescence, for example, is marked by heightened sensitivity to peer rejection; bullying during this time may have especially damaging effects. If aggression is repeatedly experienced, either directly or indirectly, it can potentially recalibrate normative beliefs and redefine what is considered acceptable behavior among peers.

One of the most compelling—and troubling—findings of the study is the higher rate of bullying victimization targeted at girls. Traditionally, boys are more likely to be involved in direct, physical bullying, while girls are at higher risk of relational aggression. Yet in this study, girls in the post‐conflict settings were more frequently bullied overall, suggesting a shift in the pattern typically observed in peacetime populations.

This finding raises several questions. Why are girls more targeted in this setting? There are studies showing war's destabilizing effect on family systems—through economic hardship, forced migration, and psychological distress—disproportionately impacting girls, who are also more vulnerable to violence in domestic spaces. In post‐migration settings, studies have reported an increased household tension and altered gender dynamics where aggression toward women and girls is more common. There may be a gendered dimension to perpetration itself, shaped by social learning, but research on perpetrators—particularly in war‐affected contexts—remains limited.

At the same time, it is important to acknowledge a tension within both research and policy discourse: although those experiencing forced migration are more likely to be victims of violence, there is a reluctance to speak openly about increases in aggression within post‐conflict contexts, particularly when such discussions risk being co‐opted by populist or xenophobic narratives. The fear of reinforcing antimigrant sentiment—especially in socio‐political climates where any transgression involving a refugee or asylum seeker is disproportionately politicized—has at times limited our collective willingness to examine these phenomena. I have experienced this firsthand: in a previous publication examining risk in post‐migration contexts, a reviewer recommended removing reference to increased domestic violence (Fazel & Betancourt, 2018). While we chose to retain the section in the final paper, it was notably omitted from subsequent summaries of the work. This experience reflects a broader discomfort within the field. Yet silence also carries risks. Without nuanced, empirically grounded understandings of how conflict, displacement, and resettlement intersect to shape behavior, we risk failing those most in need of support. It is precisely because these issues are complex and politically charged that they require careful, context‐sensitive research and thoughtful public engagement.

In focusing on how we can use these findings to improve outcomes, we need to be aware of the structural stressors that are present in post‐conflict and potentially post‐migration environments, especially for adolescents. Schools are likely to have experienced substantial disruption in conflict‐affected settings, from buildings being damaged to staff leaving or experiencing a range of potentially traumatic exposures themselves. Adolescents might be attending schools after a prolonged period of school closures or disruptions and potentially entering environments that are overcrowded, lack opportunities for psychosocial support, and have weakened disciplinary systems that in combination could contribute to a breakdown in feelings of safety in school. Yet none of this fully explains the uncomfortable truth that girls were more frequently targeted in this post‐conflict setting, adding insult to injury if the bullying is accompanied by experiences of social rejection.

Interventions need to be designed for scalability and sustainability, recognizing both the burdens and potential of educational settings. It is vital that responses avoid inadvertently reinforcing exclusion and instead provide integrated, multilevel interventions that engage individuals, families, and school communities. Addressing bullying in such contexts could include interpersonal skills development, community violence response frameworks, family support programs, and digital literacy initiatives. For schools in both post‐conflict and post‐migration contexts, a potentially helpful focus could be placed on fostering emotional regulation skills, which are foundational to healthy peer interactions (Silvers, 2022). Students in these settings are navigating the complex developmental tasks of adolescence while bearing the implications of growing up in a conflict‐affected society—one which, in the Ukrainian context, has since returned to active war.

Bullying in conflict‐affected communities, is not merely a peer problem—it can be a social indicator of broader systemic difficulties. If violence becomes woven into the fabric of daily life—through community exposure, family stress, digital content, and weakened institutions—it might influence norms, identities, and behaviours. This study powerfully illustrates that war shapes not only geopolitical landscapes, but also the everyday behavioural and emotional geographies of young people, and the hidden wars they carry within as well as into the classroom.

## Conflict of interest statement

The authors have declared that they have no competing or potential conflicts of interest.

## Data Availability

Data sharing not applicable to this article as no datasets were generated or analyzed.
